# Lipodystrophy: an uncommon cause of insulin resistance and young-onset diabetes

**DOI:** 10.1210/jcemcr/luag049

**Published:** 2026-04-06

**Authors:** Richa Chaturvedi, Aprajita Pradhan, Varsha Kachroo, Monika Sharma, Monika Goyal, Anu Mathew

**Affiliations:** Advance Center for Obesity, Diabetes and Endocrinology (ACODE), Indraprastha Apollo Hospital, Sarita Vihar, Delhi 110076, India; Department of Endocrinology, Max Superspecialty Hospital Dwarka, Delhi 110075, India; Department of Endocrinology, Yatharth Hospital, Faridabad, Haryana 121014, India; Department of Endocrinology, Aakash Hospital, Dwarka, Delhi 110059, India; Department of Endocrinology, Maharaja Agrasen Hospital, Punjabi Bagh, Delhi 110045, India; Department of Endocrinology, Fortis Hospital, Manesar, Gurugram, Haryana 122052, India

**Keywords:** lipodystrophy syndromes, insulin resistance, young-onset diabetes, genetic mutation, SHORT syndrome

## Abstract

Insulin resistance due to lipodystrophy syndromes is an uncommon but important cause of diabetes. It is often overlooked due to limited awareness. Standard glucose-lowering therapies often fail to achieve durable metabolic control in patients with lipodystrophy. Long-term complications include cardiovascular events, pancreatitis, kidney failure, metabolic dysfunction associated steatotic liver disease, and sepsis. We report a case of a 15-year-old boy presenting with darkening of skin and inability to gain weight. There was no family history of diabetes or similar disorders. Laboratory evaluation showed high insulin levels, glycosylated hemoglobin (HbA1c) 6.6% (SI: 49 mmol/mol) (reference range, < 5.7% [SI: < 39 mmol/mol]), normal lipid levels, and grade 1 fatty liver on ultrasound. He was advised on a diabetic diet and prescribed metformin; subsequently, pioglitazone was also added. Follow-up evaluations showed persistently high insulin levels, leading us to recommend genetic analysis. Whole exome sequencing revealed a heterozygous pathogenic variant in exon 15 of the regulatory subunit of phosphoinositide 3-kinase 1 (*PIK3R1*) gene, leading to a genetic diagnosis of SHORT syndrome (short stature, joint hyperextensibility, ocular depression, rieger anomaly, and teething delay). This case underscores the importance of considering lipodystrophy syndromes in lean individuals with diabetes, insulin resistance, and dysmorphic features.

## Introduction

The lipodystrophy syndromes are a heterogeneous group of rare disorders characterized by selective deficiency of adipose tissue, in the absence of nutritional deprivation or a catabolic state [1]. Lipodystrophy syndromes generally present with metabolic abnormalities associated with severe insulin resistance that include diabetes mellitus, hypertriglyceridemia, hepatic steatosis, and acanthosis nigricans. Achieving glycemic control in many patients with lipodystrophy may be difficult, although episodes of diabetic ketoacidosis are rare [2]. Lipodystrophy syndromes are frequently associated with hormonal and metabolic derangements resulting in severe comorbidities. Many complications of lipodystrophy are secondary to reduced adipose mass, leading to ectopic lipid storage in the liver, muscle, and other organs and causing insulin resistance. The diagnosis of lipodystrophy is often delayed due to the rarity of these syndromes. Establishing the subtype of lipodystrophy helps in guiding management. Current therapies prevent or ameliorate the comorbidities of lipodystrophy syndromes. Currently, there is no cure for lipodystrophy and no treatment that can regenerate adipose tissue [1].

## Case presentation

A 15-year-old boy of Asian ethnicity presented in the endocrine clinic with gradually progressive darkening of the skin, especially in the neck area, and an inability to gain weight since early childhood despite taking an age-appropriate diet. He is the only child of a nonconsanguineous marriage. His mother reports no history of using any nonprescription medications during pregnancy, obstetric complications, delayed milestones for the patient, or significant illness during his childhood. Information on birth weight and length was unavailable. He has consistently performed well academically.

There is no family history of a similar disorder, diabetes, or any significant disease.

**Figure 1:**
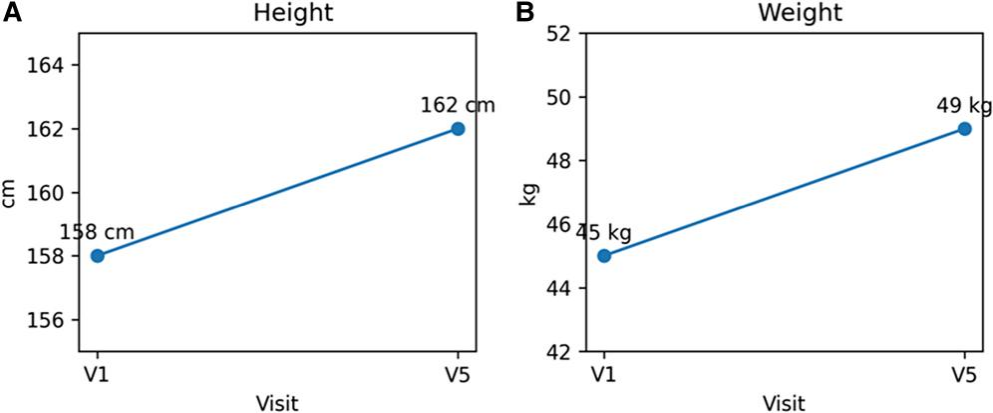
Two-panel plot of sequential anthropometric changes: (A) Height 158→162 cm [V1 (visit 1, month 0) to V5 (visit 5, month 15)]; (B) weight 45→49 kg [V1-V5].

Patient reported no history of osmotic symptoms. On examination, his height was 158 cm (25th centile according to the WHO 2006/Indian Academy of Pediatrics (IAP)2015 combined growth charts: −0.82 SDS), and his weight was 45 kg (between 10th and 25th centiles (Fig. 1). The mid-parental height was 170.5 cm. He had a triangular, progeroid facies with loss of subcutaneous fat mainly in the face, trunk, and shoulders, broad forehead, and deep-set eyes. Secondary delay in the eruption of permanent teeth was noted. Acanthosis nigricans was extensively present over the neck, elbows, knees, and skinfolds. There was no mucosal pigmentation. His blood pressure was 110/80 mmHg, pulse rate 90 beats per minute, and respiratory rate 22 breaths per minute. Examination of the neurological, cardiovascular, and gastrointestinal systems was unremarkable. He was prepubertal, with absent facial hair and no deepening of voice. The stretched penile length (SPL) measured 6.0 cm, and testicular volume (TV) assessed by the Prader orchidometer was 4 mL.

## Diagnostic assessment

Initial laboratory investigations (Table 1) showed high insulin levels. Fasting insulin level was 121.7 µIU/mL (SI: 730.2 pmol/L) (reference range, 1.9-23 µIU/mL [SI: 11.4-138 pmol/L]). 2-hour postprandial (PP) insulin level, following a diet of rice and fish curry, was 172.6 µIU/mL (SI: 1035.6 pmol/L) (reference range, 22-75 µIU/mL [SI: 132-450 pmol/L]). Fasting and 2-hour PP blood glucose levels were 141 mg/dL (SI:7.8 mmol/L) (reference range, 71-99 mg/dL [SI: 3.9-5.5 mmol/L]), and 239 mg/dL (SI: 13.3 mmol/L) (reference range, 141-199 mg/dL [SI: 7.8-11.1 mmol/L]), respectively. Glycosylated hemoglobin (HbA1c) value was 6.6% (SI: 49 mmol/mol). Lipid profile showed total cholesterol 113.4 mg/dL (SI: 2.9 mmol/L) (reference range, 120-200 mg/dL [SI: 3.1-5.2 mmol/L]), triglyceride (TG) 96.7 mg/dL (SI: 1.09 mmol/L) (reference range, < 200 mg/dL [SI:<2.26mmol/L]), high-density lipoprotein (HDL) 32 mg/dL (SI: 0.83 mmol/L) (reference range, > 55 mg/dL [SI: > 1.4 mmol/L]), and low-density lipoprotein (LDL) 65 mg/dL (SI: 1.70 mmol/L) (reference range, < 100 mg/dL [SI:<2.6 mmol/L]). Creatinine (Cr) was within normal limit (0.9 mg/dL [SI: 80 µmol/L]) (reference range, 0.7-1.2 mg/dL [61.9-106.1 µmol/L]). Relevant hormonal investigations for delayed puberty were advised but not done. Serum adiponectin and leptin levels were not measured. All investigations during visit 1 were performed at our endocrine clinic, whereas subsequent investigations were conducted at his native place in a different city. The patient lives and studies in a different city, so he came in person only for visit 1. His mother attended follow-up visits 2, 3, 4, and 5 with reports.

**Table 1:** Laboratory investigations

Parameter	Visit 1 (month 0)	Visit 2 (month 1)	Visit 3 (month 7)	Visit 4 (month 14)	Visit 5 (month 15)	Reference range
Insulin, fasting						
Conventional (SI)	121.7 µIU/mL (730.2 pmol/L)	154.8 µIU/mL (928.8 pmol/L)	48.04 µIU/mL (288.24 pmol/L)	239 µIU/mL (1434 pmol/L)		1.9-23 µIU/mL (11.4-138 pmol/L)
Insulin, 2-hour postprandial						
Conventional (SI)	172.6 µIU/mL (1035.6 pmol/L)	391.7 µIU/mL (2350.2 pmol/L)	282.6 µIU/mL (1695.6 pmol/L)	>1000 µIU/mL (>6000 pmol/L)		22-75 µIU/mL (132-450 pmol/L)
HbA1c						
Conventional (SI)	6.6% (49 mmol/mol)	6.0% (42 mmol/mol)	5.7% (39 mmol/mol)			<5.7% (<39 mmol/mol)
Fasting blood glucose						
Conventional (SI)	141 mg/dL (7.8 mmol/L)	104 mg/dL (5.8 mmol/L)	85 mg/dL (4.7 mmol/L)	86 mg/dL (4.8 mmol/L)		71-125 mg/dL (3.9-5.5 mmol/L)
2-hour postprandial blood glucose						
Conventional (SI)	239 mg/dL (13.3 mmol/L)	100.8 mg/dL (5.6 mmol/L)	150 mg/dL (8.3 mmol/L)	122 mg/dL (6.8 mmol/L)		71-140 mg/dL (7.8-11.1 mmol/L)
Creatinine						
Conventional (SI)	0.9 mg/dL (80 µmol/L)			0.53 mg/dL (47.1 µmol/L)		0.7-1.2 mg/dL (61.9-106.1 µmol/L)
Total cholesterol						
Conventional (SI)		113.4 mg/dL (2.9 mmol/L)				120-200 mg/dL (3.1-5.2 mmol/L)
Triglyceride						
Conventional (SI)		96.7 mg/dL (1.09 mmol/L)				<200 mg/dL (< 2.26 mmol/L)
HDL						
Conventional (SI)		32 mg/dL (0.83 mmol/L)				>55 mg/dL (> 1.4 mmol/L)
LDL						
Conventional (SI)		65 mg/dL (1.70 mmol/L)				<100 mg/dL (<2.6 mmol/L)
VLDL						
Conventional (SI)		19.34 mg/dL (0.22 mmol/L)				10-40 mg/dL (0.11-0.45 mmol/L)
Ultrasound abdomen	Grade I fatty liver (normal size with increased echogenicity)					
Hemoglobin						
Conventional (SI)				12.6 g/dL (126 g/L)		13-17 g/dL (130-170 g/L)
Total leukocyte count						
Conventional (SI)				9200 per µL (9.2 × 10^9^ per L)		4000-10 000 per µL (4-10 × 10^9^ per L)
Platelet count						
Conventional (SI)				334 000 per µL (334 × 10^9^ per L)		150 000-400 000 per µL (150-400× 10^9^ per L)
ESR						
Conventional (SI)				10 mm/hour (10 mm/hour)		0-10 mm/hour (0-10 mm/hour)
Whole exome sequencing					*PIK3R1* pathogenic variant. Variation chr5:67592129C > T c.1945C > T p.Arg649Trp	

Abbreviation: ESR, erythrocyte sedimentation rate; HDL, high-density lipoprotein; VLDL, very low density lipoprotein.

**Figure 2:**
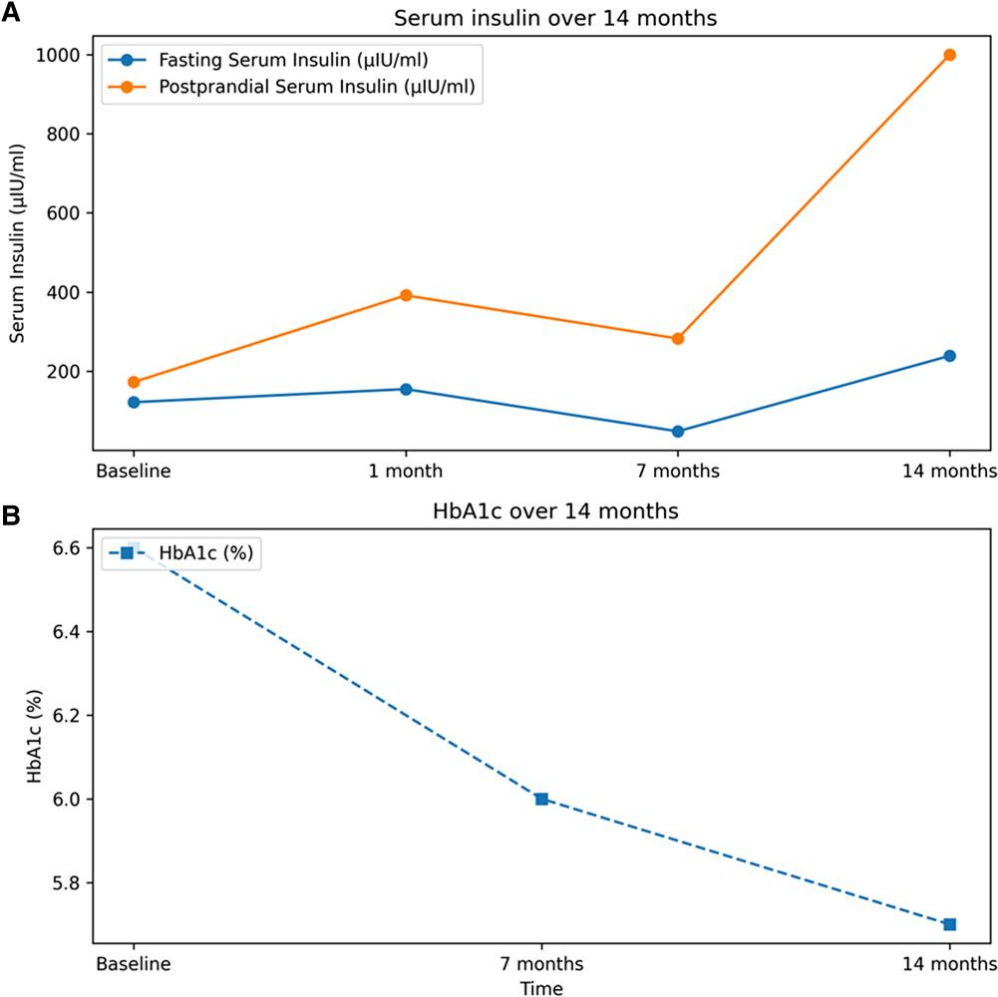
(A) Serum insulin over 14 months, (B) HbA1c over 14 months. Notes: Postprandial insulin value at 14 months was reported as >1000 µIU/mL and is graphically capped at 1000 for visualization. HbA1c values are plotted at baseline, 7 months, and 14 months. Metformin 1000 mg/day was started at baseline. Pioglitazone 15 mg/day was added at 7 months.

Sequential investigation reports are detailed in Table 1. Sequential HbA1c and insulin levels are depicted in a graph in Fig. 2. Continuous glucose monitoring was not done. Ultrasound of the abdomen showed grade 1 fatty liver with increased echogenicity.

**Figure 3:**
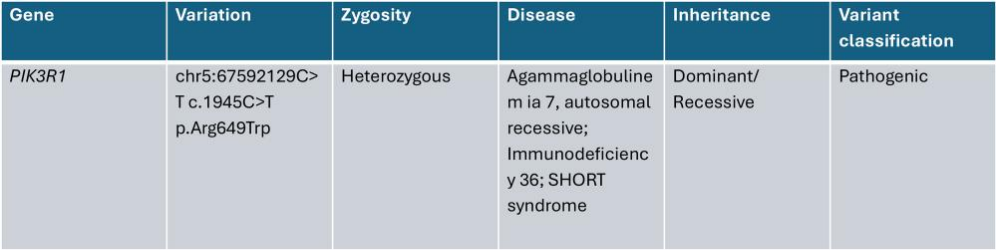
Whole exome sequencing. Key findings.

Whole exome sequencing was advised on visit 4 after discussion with the mother, which revealed the presence of a heterozygous pathogenic variant in exon 15 of the *PIK3R1* gene and a diagnosis of SHORT syndrome (short stature, hyperextensibility, hernia, ocular depression, rieger anomaly, teething delay, facial gestalt consisting of triangular facies, lack of facial fat, hypoplastic nasal alae with overhanging columella, partial lipodystrophy, insulin resistance, nephrocalcinosis, and hearing deficits; Fig. 3).

Based on clinical features and biochemical findings, a provisional diagnosis of insulin-resistant diabetes was made.

## Treatment

The patient was counseled about the diabetic diet and exercise and was started on metformin 1000 mg daily. His blood glucose levels improved with metformin, but insulin levels and leanness did not. At visit 3, fasting and 2-hour PP insulin levels were 48.04 µIU/mL (288.24 pmol/L) and 282.6 µIU/mL (1695.6 pmol/L), respectively. HbA1c was 5.7% (39 mmol/mol; Fig. 2). Therefore, at his third consultation, 7 months after the initial presentation, pioglitazone 15 mg per day was added to metformin 1000 mg per day. Pioglitazone use was off-label given the patient's age. Follow-up reports 7 months after combined metformin and pioglitazone use showed rising insulin levels with reasonable glycemic control (Fig. 2). Very high insulin levels despite treatment with insulin sensitizers led us to investigate a possible monogenic cause.

## Outcome and follow-up

The discordance between rising insulin concentrations and stable glycemic indices prompted evaluation for a monogenic cause of insulin resistance.

Following the detection of a *PI3KR1* gene mutation, the family was briefed on the future implications and the need for regular medical checkups, including eye examinations, intraocular pressure measurements, and hearing tests every 2 to 3 years. Genetic counseling was discussed, explaining that each child of an individual with SHORT syndrome has a 50% chance of inheriting the pathogenic variant [3]. Metformin and pioglitazone were continued. At visit 5, his height was reported as 162 cm (between the 10th and 25th percentile, −0.75 SDS) and his weight as 49 kg (at the 25th percentile; Fig. 1). Facial hair on the upper lip and along the sides of the cheeks was also reported.

## Discussion

This case highlights SHORT syndrome due to a pathogenic heterozygous *PIK3R1 c.1945C*  *>*  *T (p.Arg649Trp)* variant as the unifying diagnosis underlying severe insulin resistance, marked hyperinsulinemia, partial lipodystrophy, early-onset diabetes, and dysmorphic facial features in a lean adolescent. The characteristic facial gestalt differentiates SHORT syndrome from most other genetic lipodystrophies. Individuals with Silver Russell Syndrome may share some facial features, but they have significant short stature and do not have lipodystrophy.

The *PIK3R1* gene encodes the regulatory subunit of phosphoinositide 3-kinase (PI3K), which plays a key role in insulin signaling through binding of phosphorylated insulin-receptor substrates, production of phosphatidylinositol-4,5-trisphosphate (PIP3), and subsequent activation of AKT serine-threonine kinase (also known as protein kinase B), crucial for cell growth, proliferation, and survival [4]. Pathogenic variants, as found in our patient, impair PI3K–AKT pathway activation, resulting in post-receptor insulin resistance (proximal to Akt) with preserved or exaggerated β-cell secretory capacity.

It is pertinent here to recall the proposed model of hepatic lipogenesis in the commonly occurring form of insulin resistance, wherein selective post-receptor hepatic insulin resistance is implicated. According to this model, the canonical insulin signaling pathway through insulin receptor substrate/PI3K/AKT/forkhead box O transcription factor 1 (IRS/PI3 K/AKT/FOXO1) is selectively downregulated in the presence of hyperinsulinemia. In contrast, a parallel pathway linking activation of the insulin receptor to transcriptional upregulation of the critical lipogenic transcription factor SREBP1c remains fully functional and thus mediates enhanced lipogenesis in the presence of hyperinsulinemia [5].

The clinical syndrome associated with *PIK3R1* mutation that we describe is consistent with previous reports of SHORT syndrome, except for the finding of fatty liver. It has previously been shown that patients with insulin receptor (*INSR*) mutations, despite severe insulin resistance, do not show fatty liver, dyslipidemia, or suppressed plasma adiponectin, whereas patients with severe insulin resistance ascribed to an AKT2 mutation exhibit all of these to a severe degree. The insulin resistance subphenotype of SHORT syndrome patients, who have a functional defect between INSR and AKT2 in the insulin signaling pathway, closely resembles that seen in INSR dysfunction [4]. Our finding of normolipidemic severe insulin resistance with fatty liver may indicate a proximal insulin signaling defect with a small increase in hepatic lipogenesis due to some residual post-receptor PI3K signaling capacity in hepatocytes.

Liver fat measurements by a more sensitive method like proton magnetic resonance spectroscopy would better inform about the magnitude of hepatic lipogenesis. The magnitude of hepatic lipogenesis is remarkably small in comparison with the huge increases in plasma insulin.

Normolipidemia in the presence of grade 1 hepatic steatosis represents another intriguing feature. In this setting, hepatic steatosis likely reflects ectopic lipid accumulation driven by impaired adipose storage capacity and intrinsic hepatic insulin signaling defects, rather than systemic lipid oversupply. Other undetected modifier genes may also contribute to the varying clinical presentations in different patients [6]. There may also be decreased oxidation of hepatic TGs, resulting in normolipidemia, although this cannot be confirmed.

Serum adiponectin was not measured in this patient, which represents a limitation. Reduced adiponectin levels are commonly described in lipodystrophy and may further exacerbate hepatic insulin resistance and intrahepatic lipid accumulation despite preserved plasma lipid profiles [7].

The observed increase in insulin levels after initiating pioglitazone is paradoxical, particularly given the patient's stable body weight and acceptable glycemic control. This phenomenon warrants further investigation. It is possible that the onset of puberty has played a role in this change, as indicated by the appearance of facial hair. Puberty is characterized by transient reductions in insulin sensitivity driven by growth hormone and sex steroid exposure, and this effect is likely amplified in individuals with preexisting defects in insulin signal transduction. However, we are unable to comment on TV, SPL, or pubic hair development, as the patient was not present during the visit.

One report observed a worsening of insulin resistance and glucose tolerance in a child with SHORT syndrome treated with metformin. The authors speculated that metformin might further inhibit *PIK3R1* signaling [8].

So far, fewer than 50 individuals have been found to carry a pathogenic variant in *PIK3R1*. To our knowledge, this is the first case report of this mutation in India.

## Learning points

- Severe insulin resistance in lean individuals should prompt evaluation for lipodystrophy syndromes.
- SHORT syndrome should be considered when partial lipodystrophy coexists with normolipidemia, disproportionate hyperinsulinemia, and dysmorphic features.
- Genetic diagnosis helps in the interpretation of biochemical responses, treatment, and prognostication.

## Data Availability

Some or all datasets generated during and/or analyzed during the current study are not publicly available but are available from the corresponding author on reasonable request.

## References

[1] Brown RJ, Araujo-Vilar D, Cheung PT, et al The diagnosis and management of lipodystrophy syndromes: a multi-society practice guideline. J Clin Endocrinol Metab. 2016;101(12):4500‐4511. 27710244 10.1210/jc.2016-2466 PMC5155679

[2] Robbins DC, Sims EA. Recurrent ketoacidosis in acquired, total lipodystrophy (lipoatrophic diabetes). Diabetes Care. 1984;7(4):381‐385. 6432502 10.2337/diacare.7.4.381

[3] Innes AM, Dyment DA. SHORT syndrome. 2014 May 15 [Updated 2020 Jun 4]. In: Adam MP, Bick S, Mirzaa GM, et al, editors. GeneReviews® *[Internet]*. University of Washington, Seattle; 1993-2025.

[4] Dyment DA, Smith AC, Alcantara D, et al Mutations in PIK3R1 cause SHORT syndrome. Am J Hum Genet. 2013;93(1):158‐166. 23810382 10.1016/j.ajhg.2013.06.005 PMC3710754

[5] Semple RK, Sleigh A, Murgatroyd PR, et al Postreceptor insulin resistance contributes to human dyslipidemia and hepatic steatosis. J Clin Invest. 2009;119(2):315‐322. 19164855 10.1172/JCI37432 PMC2631303

[6] Lewandowski KC, Dąbrowska K, Brzozowska M, Kawalec J, Lewiński A. Metformin paradoxically worsens insulin resistance in SHORT syndrome. Diabetol Metab Syndr. 2019;11(1):81. 31583022 10.1186/s13098-019-0477-z PMC6771105

[7] Huang-Doran I, Tomlinson P, Payne F, et al Insulin resistance uncoupled from dyslipidemia due to C-terminal PIK3R1 mutations. JCI Insight. 2016;1(17):e88766. 27766312 10.1172/jci.insight.88766 PMC5070960

[8] Chudasama KK, Winnay J, Johansson S, et al SHORT syndrome with partial lipodystrophy due to impaired phosphatidylinositol 3 kinase signaling. Am J Hum Genet. 2013;93(1):150‐157. 23810379 10.1016/j.ajhg.2013.05.023 PMC3710758

